# 

**Published:** 1893-10-14

**Authors:** 


CONTENTS.

The Dead or the Living? - 17
Around the Hospitals - 18
Some Modern Aspects of Diabetes. II - 19
" A New Method of Treating the Insane " - 20
A Physiological Lantern. Who creates Criminal Children? - 21
A Case for Medical Discipline - 22
Notes and News - 24
The Hospital Clinic—
St. Mary's Hospital, London.—On the Treatment of Otorrhœa in the Aural Department - 25
The Treatment of Ringworm at Bristol General Hospital and Elsewhere - 26
New Drugs and Preparations.—Cocaine in Ophthalmic Work - 27
Annotations.—Cremation at the Church Congress—Cancer Researches—Simple Methods in Surgery - 23
The Institutional Workshop—
Hospital Administration.—VI. Special Hospitals: Miscellaneous. Cost of Management - 29
The Lunacy Blue Book - 31
Practical Departments.—Instrument Table - 31
Construction Notes - 31
Editor's Letter-box.—The Royal Free Hospital - 32
Scraps and Gleanings - 32
The Book World of Medicine and Science - vii
Medical Vacancies and Appointments - x

EXTRA SUPPLEMENT.—THE NURSING MIRROR.

News from the Nursing World.—Our Christmas Competitions—Deadly Drugs—Unprofessional Cooks—Save us from Our Friends—Incompetent Organisers—Novel Training—Weighty Trifles—Disguised Dangers—Short Items - xi
On the Nursing of Diseases of the Nervous System.—II. Hemiplegia - xiii
Nursing in the United States.—Trained Nurses as Superintendents of Hospitals - xiv
Nursing in Paris.—IV. A Visit to the Children (*continued*) xv
News from Victoria - xv
A Quarantine Home for Nurses - xvii
Everybody's Opinion.—An Isolated Small-pox Case—London School of Medicine for Women—Nurses' Uniforms—Miss Hillyer's Corrections—District Nursing - xvi
Novelties for Nurses - xvii
Minor Appointments.—Wants and Workers - xvii
The Novel Competition.—Libraries for Nurses - xvii
Where to Go - xvii
The Muses' Looking Glass.—The Simple Adventures of a Memsahib - xviii
Appointments.—Notes and Queries - xviii
The Book World for Women and Nurses - xix

